# Case Report: *Alocasia* poisoning presenting with marked QTc prolongation and a MINOCA-like phenotype

**DOI:** 10.3389/fcvm.2026.1833677

**Published:** 2026-05-29

**Authors:** Jin-Mei Xie, Ren-Rong Lu, Zong-Jie Zheng, Jie Ma, Cheng-Hua Shan

**Affiliations:** 1 Department of Electrocardiogram Diagnosis, Sanming First Hospital Affiliated to Fujian Medical University, Sanming, China; 2 Department of Cardiovascular Medicine, Sanming First Hospital Affiliated to Fujian Medical University, Sanming, China; 3 Department of Ultrasound Medicine, Sanming First Hospital Affiliated to Fujian Medical University, Sanming, China

**Keywords:** *Alocasia* poisoning, case report, MINOCA-like presentation, QT prolongation, toxic myocardial injury

## Abstract

A 75-year-old woman accidentally ingested approximately 10 g of the rhizome of *Alocasia macrorrhizos*. Within seconds, she developed oral numbness, throat swelling, sialorrhea, dysphagia, and heartburn. She immediately rinsed her mouth with copious amounts of water and presented to a local clinic. An electrocardiogram (ECG) obtained there was normal, with a corrected QT interval (QTc) of 400 ms. Following treatment with anti-allergic therapy, her symptoms resolved. Approximately 26 h after ingestion, she developed persistent, progressive substernal chest pressure. Upon presentation to our hospital (28 h post-ingestion), her cardiac troponin I (cTnI) level was 0.352 ng/mL, while myocardial enzymes and D-dimer were within normal limits. The ECG revealed diffuse T-wave inversions and a markedly prolonged QTc interval of 590 ms. Coronary angiography showed no obstructive lesions and normal coronary flow. Echocardiography demonstrated reduced wall motion in the mid-segment of the left ventricular anterior wall, consistent with a MINOCA-like phenotype. A final diagnosis of toxic myocardial injury was made. Following supportive care, including continuous ECG monitoring, myocardial metabolic support, and rate control with low-dose metoprolol, her chest pain completely resolved approximately 124 h after ingestion. The QTc interval normalized to 440 ms, T-wave inversions became less pronounced, and regional wall motion abnormalities resolved. At a 2-week follow-up, the QTc interval was 420 ms, with further improvement in T-wave inversions.

**Conclusion:**

This case demonstrates that *Alocasia* poisoning can cause marked QTc prolongation, diffuse T-wave inversions, and reversible segmental wall motion abnormalities, presenting with a MINOCA-like phenotype. Notably, the patient exhibited significant QTc prolongation despite only mildly elevated cardiac biomarkers, suggesting that the predominant mechanism of toxic myocardial injury in this context may be electrophysiological derangement rather than structural myocyte necrosis. In patients presenting with chest pain accompanied by pronounced QTc prolongation, only mildly elevated cardiac biomarkers, and normal coronary angiography, a detailed exposure history, including inquiry about plants, should be routinely obtained, and continuous ECG monitoring is warranted.

## 1 Introduction

Chest pain accompanied by elevated cardiac biomarkers and electrocardiographic repolarization abnormalities is a common presentation in emergency departments, and acute coronary syndrome (ACS) is typically the primary consideration (1). Myocardial infarction with non-obstructive coronary arteries (MINOCA) represents an important subtype of ACS, accounting for 6%–15% of cases (2). However, certain exogenous toxins can also induce chest pain, electrocardiographic abnormalities, and myocardial injury, thereby mimicking the presentation of ischemic heart disease and increasing the risk of misdiagnosis (3).

*Alocasia macrorrhizos*, a plant belonging to the Araceae family, is widely distributed in southern China and Southeast Asia. Accidental ingestions of its rhizome are not uncommon due to its resemblance to taro (4). *Alocasia* contains various bioactive constituents, including calcium oxalate raphides, saponins, and alkaloids, which can cause oropharyngeal irritation and neurotoxicity (5). Reports of cardiac involvement following *Alocasia* poisoning are scarce. To date, only isolated studies have suggested that electrocardiographic manifestations may include left ventricular high voltage (6); however, no case has been reported with concurrent marked QTc prolongation and reversible segmental wall motion abnormalities.

We report a case of *Alocasia* poisoning presenting with chest pain, diffuse T-wave inversions, marked QTc prolongation, and reversible regional wall motion abnormalities against a backdrop of normal coronary arteries—a MINOCA-like phenotype not previously documented for this plant toxin. We report this case to highlight toxic myocardial injury as a potential etiology in such presentations and to raise awareness among clinicians in regions where *Alocasia* is prevalent.

## 2 Case presentation

A 75-year-old woman presented to the Sanming First Affiliated Hospital of Fujian Medical University on February 10, 2026, with persistent chest pain. Approximately 28 h prior to presentation, she had accidentally ingested approximately 10 g of *Alocasia macrorrhizos* rhizome. Within seconds of ingestion, she developed oral numbness, throat swelling, sialorrhea, dysphagia, and heartburn. She immediately rinsed her mouth with copious amounts of water and sought care at a local clinic, where an electrocardiogram (ECG) was normal, with a QTc interval of 400 ms (Figure 1A). Following treatment with dexamethasone, calcium gluconate, and levocetirizine for presumed allergic reaction, throat swelling resolved within approximately 4 h of treatment, while dysphagia persisted for approximately 20 h before resolving. Approximately 26 h after ingestion, she developed persistent substernal chest pressure that progressively worsened, prompting presentation to our hospital.

**Figure 1:**
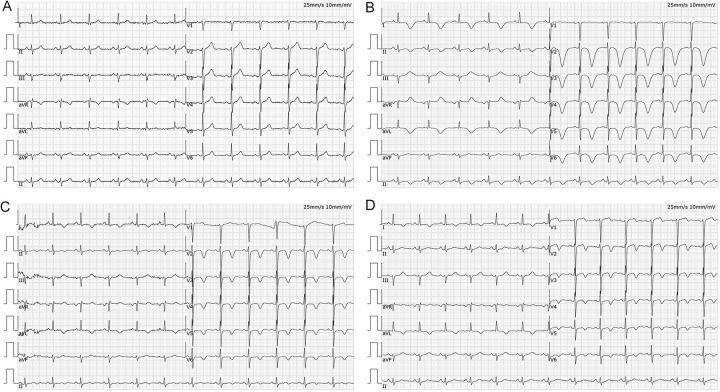
Electrocardiographic evolution. **(A)** ECG obtained at the local clinic immediately after ingestion, demonstrating normal findings with a QTc interval of 400 ms. **(B)** ECG on the first hospital day (28 h post-ingestion), showing sinus rhythm with a markedly prolonged QTc interval of 590 ms and diffuse T-wave inversions in leads I, II, aVL, and V₁ through V₆. **(C)** ECG on hospital day 5, demonstrating a decrease in the QTc interval to 440 ms and less pronounced T-wave inversions. **(D)** ECG at 2-week follow-up, showing a QTc interval of 420 ms with further improvement in T-wave inversions.

Her medical history included hypertension for 10 years, which was well-controlled with regular amlodipine therapy. She had no history of smoking or alcohol use. There was no recent infection or emotional stress, and no family history of arrhythmias or sudden cardiac death. On admission, her vital signs were stable, and physical examination was unremarkable.

Serial cTnI measurements demonstrated a peak level of 0.352 ng/mL at 28 h post-ingestion, decreasing to 0.263 ng/mL at 30 h, and normalizing to 0.015 ng/mL by 124 h when chest pain had resolved. Serum electrolyte levels were within normal limits: potassium 4.6 mmol/L, sodium 138 mmol/L, chloride 102 mmol/L, calcium 2.51 mmol/L, and magnesium 0.92 mmol/L. Myocardial enzymes, liver and renal function tests, coagulation parameters, and D-dimer (0.12 mg/L) were all normal.

### 2.1 Electrocardiography

The ECG obtained at the local clinic immediately after ingestion was normal, with a QTc interval of 400 ms (Figure 1A). On the first hospital day (28 h post-ingestion), ECG revealed sinus rhythm with a markedly prolonged QTc interval of 590 ms, diffuse T-wave inversions (2–11 mm) in leads I, II, aVL, and V₁ through V₆, and a QT dispersion of 70 ms (Figure 1B). By hospital day 5, the QTc interval had decreased to 440 ms, with less pronounced T-wave inversions (2–5 mm) in leads I, II, aVL, and V₂ through V₆ (Figure 1C). At 2-week follow-up, the QTc interval was 420 ms, with further improvement in T-wave inversions (2–3 mm) in leads I, aVL, and V₂ through V₆ (Figure 1D).

### 2.2 Echocardiography

Transthoracic echocardiography performed on the first hospital day demonstrated hypokinesis of the mid-segment of the left ventricular anterior wall, with a wall motion score index (WMSI) of 1.06 and regional wall motion amplitude of approximately 3 mm (Figure 2A). Repeat echocardiography on hospital day 5 showed complete normalization of wall motion in the affected segment, with an amplitude of approximately 7 mm and a WMSI of 1.0 (Figure 2B). Dynamic cine clips from the apical 2-chamber and apical 4-chamber views were not retained as separate video files at the time of the study; quantitative wall motion amplitude measurements are provided in lieu of video material.

**Figure 2:**
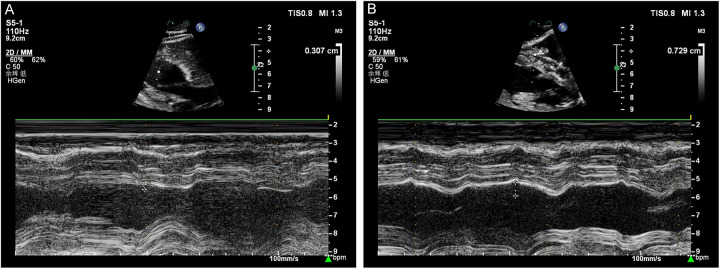
Echocardiographic findings. **(A)** Transthoracic echocardiogram obtained on the first hospital day, revealing hypokinesis of the mid-segment of the left ventricular anterior wall (arrows). **(B)** Repeat echocardiogram on hospital day 5, demonstrating complete normalization of wall motion in the affected segment.

### 2.3 Coronary angiography

Coronary angiography performed on the first hospital day revealed no obstructive stenosis in the left main, left anterior descending, left circumflex, or right coronary arteries, with Thrombolysis in Myocardial Infarction (TIMI) grade 3 flow in all vessels (Figures 3A–C).

**Figure 3:**
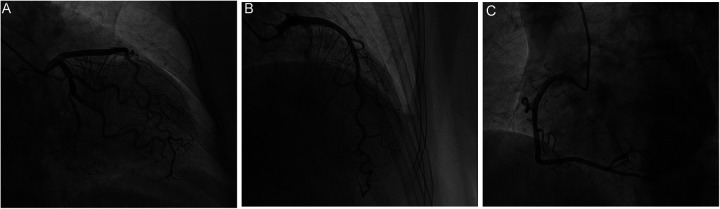
Coronary angiography. Coronary angiography performed on the first hospital day, demonstrating no obstructive stenosis in the left main, left anterior descending, left circumflex, or right coronary arteries, with TIMI grade 3 flow in all vessels. **(A)** Right anterior oblique caudal view. **(B)** Right anterior oblique cranial view. **(C)** Left anterior oblique projection.

A diagnosis of toxic myocardial injury was established clinically, though confirmatory testing was limited by delayed presentation and the unavailability of cardiac magnetic resonance (CMR). Given that the patient presented beyond the window for gastrointestinal decontamination, gastric lavage and activated charcoal were not performed. Management included continuous ECG monitoring with attention to electrolyte levels (maintaining serum magnesium >0.8 mmol/L), intravenous meglumine adenosine cyclophosphate 180 mg daily, and oral metoprolol 12.5 mg twice daily. Meglumine adenosine cyclophosphate, a cyclic adenosine monophosphate derivative, was selected as a myocardial metabolic support agent based on its established use in Chinese clinical practice; experimental evidence suggests it may attenuate myocardial remodeling by modulating the cAMP/p44/42 MAPK signaling pathway to suppress connective tissue growth factor expression and improve cardiac function indices in a murine myocardial infarction model (7). Low-dose metoprolol was initiated for rate control in the setting of marked QTc prolongation. Medications known to prolong the QT interval were avoided. Chest pain completely resolved by hospital day 5, approximately 124 h after ingestion, at which point the patient was discharged.

At 2-week follow-up, the patient remained free of chest pain, with a normal QTc interval and further improvement in T-wave inversions.

The timeline of the patient's diagnosis and treatment is shown in Figure 4.

**Figure 4:**
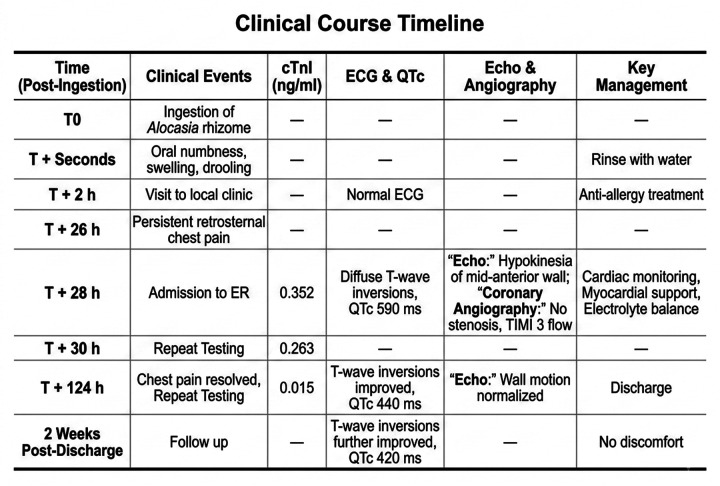
Diagnostic and treatment timeline. Timeline illustrating the patient's clinical course from ingestion through presentation, diagnosis, management, and follow-up.

## 3 Discussion

Our patient presented with chest pain, diffuse T-wave inversions, a QTc of 590 ms, and mildly elevated cTnI after accidentally ingesting *Alocasia macrorrhizos* rhizome. Coronary angiography showed no obstruction and TIMI 3 flow throughout, fulfilling the angiographic criterion for a MINOCA-like presentation. We describe this as “MINOCA-like” rather than MINOCA proper because CMR—the standard tool for identifying myocardial edema, fibrosis, or microvascular dysfunction—was not performed. Reports of cardiac involvement in *Alocasia* poisoning are exceedingly rare; prior literature mentions only left ventricular high voltage (6). To our knowledge, this is the first case documenting both pronounced electrophysiological abnormalities and reversible segmental wall motion abnormalities in this context.

The QTc reached 590 ms—well above the 500 ms threshold commonly cited as a red flag for torsade de pointes (8). Yet cTnI peaked at only 0.352 ng/mL and wall motion recovered within days. This dissociation is the most striking feature of the case: the electrical disturbance was severe; the myocyte injury, by comparison, was modest. It suggests that *Alocasia* cardiotoxicity operates primarily through ion channel disruption rather than irreversible cell death. The specific culprit compound remains unidentified, but among the bioactive constituents of *Alocasia*, alkaloids represent the most plausible candidate for the observed QTc prolongation: plant alkaloids in the Araceae family have been shown to inhibit the hERG potassium channel, which governs ventricular repolarization, a mechanism well established across multiple plant-derived alkaloid classes (9). Calcium oxalate raphides, as the primary cause of oropharyngeal injury (5), may additionally facilitate systemic entry of co-occurring alkaloids and saponins by mechanically disrupting mucosal barriers (10). It is important to note that quantitative detection of specific *Alocasia* toxins in blood or urine was not available at our institution; a direct dose-response relationship between individual toxin concentrations and the observed cardiac phenotype therefore cannot be established, and this represents a limitation of the present report. Further basic research is needed to identify the precise cardiotoxic compounds.

Several diagnoses had to be considered and then ruled out. Obstructive coronary artery disease was excluded by angiography. Takotsubo (stress-induced) cardiomyopathy could not be completely excluded: this patient was elderly, female, and the acute distress of poisoning itself may constitute a physical precipitant, all of which are recognized risk factors for Takotsubo. A mid-ventricular variant of Takotsubo, which accounts for approximately 14.6% of cases in the largest published international registry, can produce hypokinesis of the mid anterior wall rather than the classic apical ballooning pattern (11). Furthermore, electrocardiographic changes in Takotsubo may be limited to T-wave inversions without ST elevation in a subset of patients (12), consistent with the ECG findings in this case. The complete and rapid resolution of wall motion abnormalities with minimal intervention is also compatible with the transient nature of Takotsubo. Nevertheless, several features argue against this diagnosis: the markedly prolonged QTc of 590 ms, disproportionate to the mild cTnI rise, is not characteristic of Takotsubo and is better explained by a direct electrophysiological toxin effect; and the clear temporal relationship between *Alocasia* ingestion and the onset of cardiac manifestations supports a toxic etiology. We therefore favor toxic myocardial injury as the primary diagnosis, while acknowledging that Takotsubo cannot be definitively excluded in the absence of cardiac magnetic resonance imaging (12). Acute myocarditis typically announces itself with a preceding viral illness and diffuse ST elevation; neither was present here (13). Coronary spasm cannot be absolutely excluded without provocative testing, though the absence of rest chest pain and transient ST elevation makes it less likely (14). Congenital long-QT syndrome (LQTS) was ruled out by the absence of any personal or family history of arrhythmias or sudden cardiac death, together with the normalization of the QTc interval during follow-up, a course inconsistent with a fixed genetic channelopathy (15, 16). Drug-induced QT prolongation, most commonly mediated by blockade of the rapidly activating delayed rectifier potassium current (IKr) encoded by the HERG gene (17, 18), was excluded after a thorough medication review confirmed that the patient was not receiving any QT-prolonging agents. Electrolyte disturbances—particularly hypokalemia, hypomagnesemia, and hypocalcemia—are well-established triggers of QT prolongation and torsade de pointes (18, 19); however, the patient's serum potassium, magnesium, and calcium levels were persistently normal. Additional clinical factors that may prolong the QT interval, such as marked bradyarrhythmias and severe hypothermia, were also absent, as her heart rate and body temperature remained stable throughout hospitalization (18).

The practical lesson from this case is straightforward. When chest pain comes with markedly prolonged QTc but only modest cTnI rise and clean coronaries, toxic myocardial injury should be on the differential—not an afterthought. In our patient's situation, the diagnosis nearly slipped through: the initial ECG was normal, symptoms temporarily resolved, and the chest pain did not begin until 26 h post-ingestion. Had the treating physician not asked about dietary history, the link to *Alocasia* ingestion might never have been made. Routine inquiry about plant or toxin exposure, especially in regions where *Alocasia* grows, costs nothing and may prevent misdiagnosis as MINOCA or Takotsubo. Continuous ECG monitoring until QTc normalizes is essential given the arrhythmia risk at these intervals.

This study has several limitations. (1) As a single case report, the findings should be extrapolated cautiously to all cases of *Alocasia* poisoning, and definitive conclusions regarding underlying pathophysiological mechanisms or generalizable clinical patterns cannot be established. (2) Due to clinical constraints, we were unable to perform qualitative or quantitative detection of specific *Alocasia* toxins in the patient's blood or urine; consequently, we could not definitively identify the precise cardiotoxic compound(s) or establish a dose-response relationship between toxin concentration and the observed clinical phenotype. (3) Although coronary angiography revealed no obstructive lesions, acetylcholine or ergonovine provocative testing was not performed, and thus we cannot entirely exclude a potential contributory role of coronary artery spasm in the patient's chest pain and electrocardiographic abnormalities. (4) CMR imaging was not obtained, limiting non-invasive assessment of more subtle pathological changes such as myocardial edema, fibrosis, or microvascular dysfunction.

### 3.1 Patient perspective

Following the diagnosis, the patient expressed surprise that the cause of her illness was accidental ingestion of *Alocasia*; however, she reported relief upon learning that the cardiac changes were reversible. She indicated satisfaction with the treatment provided during hospitalization and noted that she remained asymptomatic at follow-up, expressing confidence in her complete recovery. The patient provided consent for her clinical details to be published anonymously, with the hope of contributing to medical knowledge and improving awareness of this type of poisoning.

## 4 Conclusion

This case demonstrates that *Alocasia* poisoning can produce marked QTc prolongation, diffuse T-wave inversions, and reversible segmental wall motion abnormalities that together constitute a MINOCA-like phenotype. The striking dissociation between severe electrical disturbance—exemplified by a QTc of 590 ms—and only mildly elevated cardiac biomarkers suggests that the predominant mechanism of cardiotoxicity may be electrophysiological derangement rather than structural myocyte necrosis. Clinicians practicing in regions where *Alocasia* is prevalent should maintain a high index of suspicion for toxic myocardial injury in patients presenting with chest pain, pronounced QTc prolongation, modest cTnI elevation, and non-obstructive coronary arteries. A thorough toxicological and exposure history, including inquiry about plant ingestion, should be routinely obtained, and continuous ECG monitoring is warranted until the QTc interval normalizes. Given the rarity of such presentations, further multi-center case documentation and basic research are needed to identify the specific cardiotoxic compounds and establish evidence-based management strategies.

## Data Availability

The original contributions presented in the study are included in the article/Supplementary Material, further inquiries can be directed to the corresponding authors.
